# Association between sperm DNA fragmentation index and recurrent pregnancy loss: results from 1485 participants undergoing fertility evaluation

**DOI:** 10.3389/fendo.2024.1493186

**Published:** 2025-01-07

**Authors:** Guanying Yao, Xianchao Dou, Xiaozhu Chen, Haolin Qi, Jianling Chen, Peiwei Wu, Jialu Li, Shuang Liang, Zhongjiang Han, Shun Bai, Xu Hu

**Affiliations:** ^1^ Department of Reproductive Medicine, Nanyang Central Hospital, Nanyang, China; ^2^ School of Medical Imaging, Bengbu Medical University, Bengbu, China; ^3^ School of Nursing, Anhui University of Chinese Medicine, Hefei, China; ^4^ Sydney School of Public Health, Faculty of Medicine and Health, University of Sydney, Camperdown, NSW, Australia; ^5^ Center for Reproduction and Genetics, The First Affiliated Hospital of USTC, Division of Life Sciences and Medicine, University of Science and Technology of China, Hefei, China

**Keywords:** DNA fragmentation index, recurrent pregnancy loss, semen quality, DFI, RPL

## Abstract

**Objective:**

Several male factors have been reported to play a role in recurrent pregnancy loss (RPL). The aim of this study is to explore the relationship between semen parameters, sperm DNA fragmentation index (DFI) and RPL.

**Method:**

A total of 1485 participants were recruited from a university hospital between April 2020 and August 2022. Six hundred and thirtyfour men from couples with RPL were assigned to the case group, while 851 men from couple without RPL who underwent fertile evaluation were assigned to the control group. Semen parameters including sperm DNA fragmentation, were assessed.

**Results:**

No statistically significant differences in semen parameters, sperm kinematics and DFI were observed between the case group and the control group. A higher proportion of men in the case group had a DFI > 30% compared to those in the control group; however, this difference was not statistically significant. Restricted cubic spline analysis revealed no significant non-linear relationships between continuous DFI and risk of RPL.

**Conclusion:**

Our study indicates that there is no significant relationship between DFI and RPL risk. Further prospective studies are needed to explore the impact of DFI on fertility outcomes in couples experiencing RPL.

## Introduction

Approximately 15% of reproductive-age couples suffer from infertility globally, with male factors accounting for half of these cases (1, 2). Conventional semen analysis is a standard method for evaluating semen quality, including parameters such as volume, sperm concentration, motility and morphology. However, this method is limited to evaluating basic parameters and additional sperm functions, such as DNA integrity, acrosome reaction and capacitation, require further assessment. Sperm DNA integrity is a critical parameter for male reproduction. Due to the complex organization of haploid genome, the DNA within the sperm nucleus is often altered by protamine-mediated compaction (3). Previous studies have demonstrated that sperm DNA fragmentation can adversely affect fertilization and embryo development (4–6). DNA Fragmentation Index (DFI) is a parameter used to assess the integrity of sperm DNA. Specifically, DFI measures the percentage of spermatozoa exhibiting DNA fragmentation, which can be an indicator of sperm quality and fertility potential. High DFI values are associated with increased sperm DNA damage, which may result from factors like oxidative stress, apoptosis, or autophagy (7).

Recurrent pregnancy loss (RPL) is defined as two or more clinically recognized pregnancy losses before 20-24 weeks of gestation, including both embryo and fetal loss (8). According to previous epidemiological studies, the prevalence of RPL is 1-4% among all patients who achieve pregnancy (9). Several factors have been suggested to contribute to pathogenesis of RPL, including maternal age, genetic defects, uterine pathology, endocrine disorders, and infectious agents. However, the exact etiology of RPL in approximately 50-75% of patients remains unclear (10). Although most known causes of RPL are related to female factors, recent studies have also indicated that semen quality may influence the risk of PRL. A systematic review and meta-analysis including 16 studies and 2969 couples suggested that increased DFI was associated with an increased risk of RPL (11). Additionally, several studies reported male partners of couples with RPL were more likely to have high DFI compared with those with low DFI (12, 13). Importantly, new guidelines from the European Society of Human Reproduction and Embryology (ESHRE), the American Urological Association (AUA), and the American Society for Reproductive Medicine (ASRM) recommend sperm DFI as a parameter for evaluating RPL (14–16).

Assays for analyzing sperm DNA integrity have been developed in recent years, including the terminal deoxynucleotidyl transferase dUTP nick end labeling (TUNEL) assay, the comet assay, the sperm chromatin dispersion (SCD) assay, and the sperm chromatin structure assay (SCSA). In a recent review, the authors provided a comprehensive summary of the techniques, advantages, and disadvantages of the TUNEL, SCSA, SCD, and Comet assays (17). The terminal deoxynucleotidyl transferase-mediated deoxyuridine triphosphate nick end labeling (TUNEL) assay is noted for its sensitivity, reliability, and minimal interrater variability. However, its disadvantages include high cost, the need for specialized equipment, and extensive training requirements. The sperm chromatin dispersion (SCD) assay is advantageous due to its simplicity and minimal equipment requirements, though it is hindered by a high degree of interobserver variability. The comet assay offers sensitivity and reproducibility, but suffers from interobserver variability, the necessity for an experienced operator, and the use of variable protocols. Finally, the sperm chromatin structure assay (SCSA) is characterized by a standardized protocol, reproducibility, and the ability to examine a large number of cells, though it also faces limitations in terms of high cost, the need for specialized equipment, and specific training requirements. Among these tests, SCSA is utilized for assessing the DFI in several large cohorts, although there is an ongoing effort to establish a consensus approach that is both highly reliable and cost-effective. Date from SCSA indicate that DFI has a strong positive correlation with pregnancy outcomes and miscarriage (18). In this study, which includes a relatively large sample size, we measured sperm DFI using SCSA to investigate the association between DFI and RPL risk.

## Methods

### Participants

Between April 2020 and August 2022, a total of 1485 male partners of infertile couples at the Reproductive Center of The First Affiliated Hospital of University of Science and Technology of China (USTC) and the female partners aged between 22 and 40 years, were included in this retrospective study. Couples who conceived their previous natural pregnancy within 1 year and experienced RPL were assigned to the case group, while couples without RPL and at least one previous pregnancy or live birth were assigned to the control group. All control group underwent fertility treatments for female factor infertility such as tubal factors. Semen parameters, including sperm DNA fragmentation were assessed, and clinical characteristics, such as demographic factors, lifestyle factors, and medical history, were collected for all participants. Couples diagnosed with causes of RPL, such as abnormal karyotypes and mullerian ducts, diabetes, hyperprolactinemia, thyroid disorders and positive for the antiphospholipid antibodies, antinuclear antibodies, lupus antibodies and anti β2 glycoprotein antibodies, were excluded from the study. The exclusion criteria in male partners of infertile couples included azoospermia, testicular cancer, cryptorchidism and genetic defects related to the male reproductive tract. This study was approved by the Ethical Committee of The First Affiliated Hospital of USTC (No. 2023-RE-196).

### Routing semen analysis

All semen sample collected at couples with previous pregnancy or live birth less than one year. Semen parameters (e.g., semen volume, sperm concentration, motility, and morphology) were assessed according to the WHO criteria (37). Ejaculates were collected by masturbation, followed by liquefaction at 37°C for at least 30 minutes. Semen parameters, including sperm concentration, progressive motility, and total motility, were analyzed using a computer-assisted sperm analysis (CASA) system (SAS-II, SAS Medical, Beijing, China). Sperm morphology was assessed following Diff-Quick staining (Anke Biotechnology, Hefei, China) using a light microscope (CX33, Olympus Corporation, Tokyo, Japan). Leukocytes were evaluated following peroxidase staining (Anke Biotechnology, Hefei, China). Antisperm antibodies (AsAs) were assessed using the mixed antiglobulin reaction (MAR) test (Anke Biotechnology, Hefei, China).

### Detection of sperm DFI

The sperm DFI was assessed using the sperm chromatin structure assay (SCSA, Celula, Chengdu, China). Semen samples were diluted with TNE buffer (Tris-HCl, NaCl, and ethylenediaminetetraacetic acid (EDTA)), followed by treatment with an acidic detergent solution (TritonX-100, NaCl, and HCl, pH 1.2). After 30 seconds, staining buffer (acridine orange, citric acid, Na2HPO4, EDTA, NaCl, pH 6.0) was added. The samples were analyzed using a flow cytometer (Celula, Chengdu, China) and a minimum of 5000 cells were examined per sample. Chromatin damage was assessed using acridine orange staining, where green fluorescence indicated double-stranded DNA and red fluorescence indicated single-stranded and denatured DNA (19).

### Statistical analysis

A systematic review and meta-analysis reported that there is a mean difference of DFI of 11% between RPL and fertile control group (20). Additionally, a recent study from China showed DFI higher than 30% in unexplained RPL group was 42% (13). By setting power at 90%, alpha error at 5%, the estimated sample size was 796 with 398 in each group. Qualitative variables were described as frequencies (percentages), and quantitative variables were described as the mean ± standard deviation (SD) if normally distributed and medians (interquartile range, IQR) if not. Pearson’s chi-square test and Student’s t test were used for parametric comparisons, and the Mann‒Whitney U test was utilized for nonparametric comparisons. Logistic regression and multivariate regression model were used to examine the association between DFI and RPL. Covariates initially included age, BMI, education, smoking, alcohol consumption, chronic diseases, urogenital infections and varicocele. Restricted cubic spline (RCS) was performed to assess for the dose-response relationships between RPL and DFI. A P value < 0.05 was considered to indicate statistical significance. All statistical analyses were performed using Prism 9.0.

## Results

Clinical characteristics of 634 men from infertile couples affected by RPL and 851 men from couples who underwent fertile evaluation are reported in **Table 1**. Age and BMI did not show any statistically significant differences between the case and control groups. Lifestyle factors, including smoking and alcohol consumption, were similar between the two groups. No significant differences were observed in educational level, sex frequency, history of chronic diseases and urogenital infections. The duration of infertility (*P* < 0.001) and the prevalence of varicocele (*P* = 0.004) were significantly higher in the case group compared to the control group.

**Table 1:** Clinical characteristics in men from the case group and the control group.

Characteristics	Total (n=1485)	Control (n=851)	Case (n=634)	*P* value
Age, mean ± s.d.	32.2 ± 5.6	32.4 ± 5.8	32.0 ± 5.4	0.29
BMI^a^, mean ± s.d.	24.7 ± 3.4	24.7 ± 3.3	24.7 ± 3.5	0.78
Smoking status, n (%)				0.13
Nonsmoker^b^	865 (58.2)	510 (59.9)	355 (56.0)	
Smoker	620 (41.8)	341 (40.1)	279 (44.0)	
Drinking status, n (%)				0.60
Nondrinker^c^	677 (45.6)	393 (46.2)	284 (44.8)	
Drinker	808 (54.4)	458 (53.8)	350 (55.2)	
Education, n (%)				0.25
Middle school or lower^d^	299 (20.1)	162 (19.1)	137 (21.6)	
High school	253 (17.1)	139 (16.3)	114 (18.0)	
College/University	933 (62.8)	550 (64.6)	383 (60.4)	
Sex frequency (weekly), n (%)				0.55
<1	174 (11.7)	93 (10.9)	81 (12.8)	
1-2	973 (65.5)	563 (66.2)	410 (64.7)	
>2	338 (22.8)	195 (22.9)	143 (22.5)	
Chronic diseases, n (%)^e^				0.27
No	1309 (88.1)	757 (89.0)	552 (87.1)	
Yes	176 (11.9)	94 (11.0)	82 (12.9)	
Duration of infertility (year), n (%)				<0.001
<1	742 (50.0)	471 (55.4)	271 (42.7)	
1-2	375 (25.2)	207 (24.3)	168 (26.5)	
>2	368 (24.8)	173 (20.3)	195 (30.8)	
Urogenital infections, n (%)^f^				0.74
No	1277 (86.0)	734 (86.3)	543 (85.7)	
Yes	208 (14.0)	117 (13.7)	91 (14.3)	
Varicocele, n (%)				0.004
No	1372 (92.4)	801 (94.1)	571 (90.1)	
Yes	113 (7.6)	50 (5.9)	63 (9.9)	

BMI, body mass index; s.d, standard deviation.

a Calculated as weight in kilograms divided by height in meters squared.

b included never smoking and no smoking during the past 3 months.

c included never drinking and no drinking during the past 3 months.

d included primary school and junior high school.

e included diabetes, hypertension, and hyperlipidemia.

f included epididymitis, prostatitis, balanoposthitis, and seminal vesiculitis.

Semen parameters were further compared between two groups. No statistically significant differences in semen volume, sperm concentration, progressive motility, total motility, TMSC, morphology, AsAs, DFI, HDS and leukocytes were found between men in the case group and those in the control group (**Table 2**). In addition, the proportion of men with abnormal semen parameters did not differ significantly between the two groups. A higher proportion of men with DFI > 30% was observed in the case group compared to the control group (6.3% vs. 5.2%), but this difference did not reach statistical significance. Similarly, the study groups were comparable in terms of sperm kinematics, including VCL, VSL, VAP, BCF, ALH, MAD, LIN, WOB, and STR (**Table 3**).

**Table 2:** Semen parameters in men from the case group and the control group.

Semen parameters	Total (n=1485)	Control (n=851)	Case (n=634)	*P* value
Volume (ml), median (IQR)	3.2 (2.3-4.2)	3.2 (2.2-4.3)	3.1 (2.3-4.1)	0.87
< 1.5 ml, n (%)	107 (7.2)	69 (8.1)	38 (6.0)	0.12
Concentration (million/ml), median (IQR)	68.2 (38.0-120.0)	67.7 (37.3-123.2)	69.5 (39.6-118.3)	0.73
< 15 million/ml, n (%)	96 (6.5)	53 (6.2)	43 (6.8)	0.67
Progressive motility (%), median (IQR)	40.4 (28.6-52.3)	39.4 (27.3-52.0)	42.1 (29.8-52.7)	0.09
< 32%, n (%)	471 (31.7)	285 (33.4)	186 (29.3)	0.09
Total motility (%), median (IQR)	45.6 (32.6-58.6)	44.5 (31.5-58.5)	47.7 (33.6-58.8)	0.12
< 40%, n (%)	577 (38.9)	346 (40.7)	231 (36.4)	0.10
TMSC (million), median (IQR)	94.9 (39.1-204.0)	92.0 (36.1-202.4)	97.0 (44.5-205.2)	0.33
< 9 million, n (%)	91 (6.1)	53 (6.2)	38 (6.0)	0.85
Normal morphology (%), median (IQR; n)	7.0 (5.0-8.0; 1437)	7.0 (5.0-8.0; 826)	7.0 (5.0-8.0; 611)	0.38
< 4%, n (%)	106 (7.4)	61 (7.4)	45 (7.4)	0.99
Leukocytes(×10^6^/ml), median (IQR; n)	0.08 (0.03-0.29; 873)	0.04 (0.01-0.08; 511)	0.06 (0.03-0.28; 362)	0.17
AsAs (%), median (IQR; n)	1.0 (0.0-3.0; 575)	1.0 (0.0-3.0; 334)	2.0 (0.0-4.0; 241)	0.20
DFI (%), median (IQR)	11.9 (7.6-17.9)	11.9 (7.6-17.8)	11.9 (7.7-18.3)	0.41
>30%, n (%)	84 (5.7)	44 (5.2)	40 (6.3)	0.35
HDS (%), median (IQR)	6.6 (4.9-8.8)	6.6 (4.8-8.8)	6.6 (5.0-8.9)	0.34

IQR, interquartile range; TMSC, total motile sperm count; AsAs, antisperm antibodies; DFI, DNA fragmentation index; HDS, high DNA stainability.

**Table 3:** Sperm kinematics in men from the case group and the control group.

Sperm kinematics	Total (n=1485)	Control (n=851)	Case (n=634)	*P* value
VCL (μm/sec), median (IQR)	32.5 (23.3-43.9)	32.0 (22.7-43.5)	33.3 (24.0-44.7)	0.18
VSL (μm/sec), median (IQR)	13.7 (9.1-18.5)	13.2 (8.8-18.5)	14.2 (9.6-18.6)	0.19
VAP (μm/sec), median (IQR)	17.5 (12.0-23.5)	16.9 (11.7-23.4)	18.1 (12.6-23.7)	0.18
BCF (Hz), median (IQR)	8.3 (5.7-11.6)	8.0 (5.4-11.6)	8.7 (5.9-11.6)	0.13
ALH (μm/sec), median (IQR)	2.3 (2.0-2.4)	2.2 (2.0-2.4)	2.3 (2.0-2.5)	0.25
MAD (degrees), median (IQR)	13.9 (11.1-16.6)	13.7 (10.9-16.6)	13.9 (11.3-16.7)	0.30
LIN, median (IQR)	38.7 (32.8-43.9)	38.4 (32.5-43.8)	39.1 (33.2-44.2)	0.26
WOB, median (IQR)	50.8 (45.3-55.1)	50.6 (45.1-55.0)	51.2 (45.5-55.4)	0.25
STR, median (IQR)	74.8 (64.4-79.4)	74.4 (63.9-79.2)	75.3 (65.1-79.6)	0.14

VCL, curvilinear velocity; VSL, straight-line velocity; VAP, average path velocity; BCF, frequency of beat cross; ALH, mean amplitude of lateral head displacement; MAD, mean angular displacement; LIN, linearity coefficient; WOB, wobble coefficient; STR, straightness coefficient.

**Figure 1:**
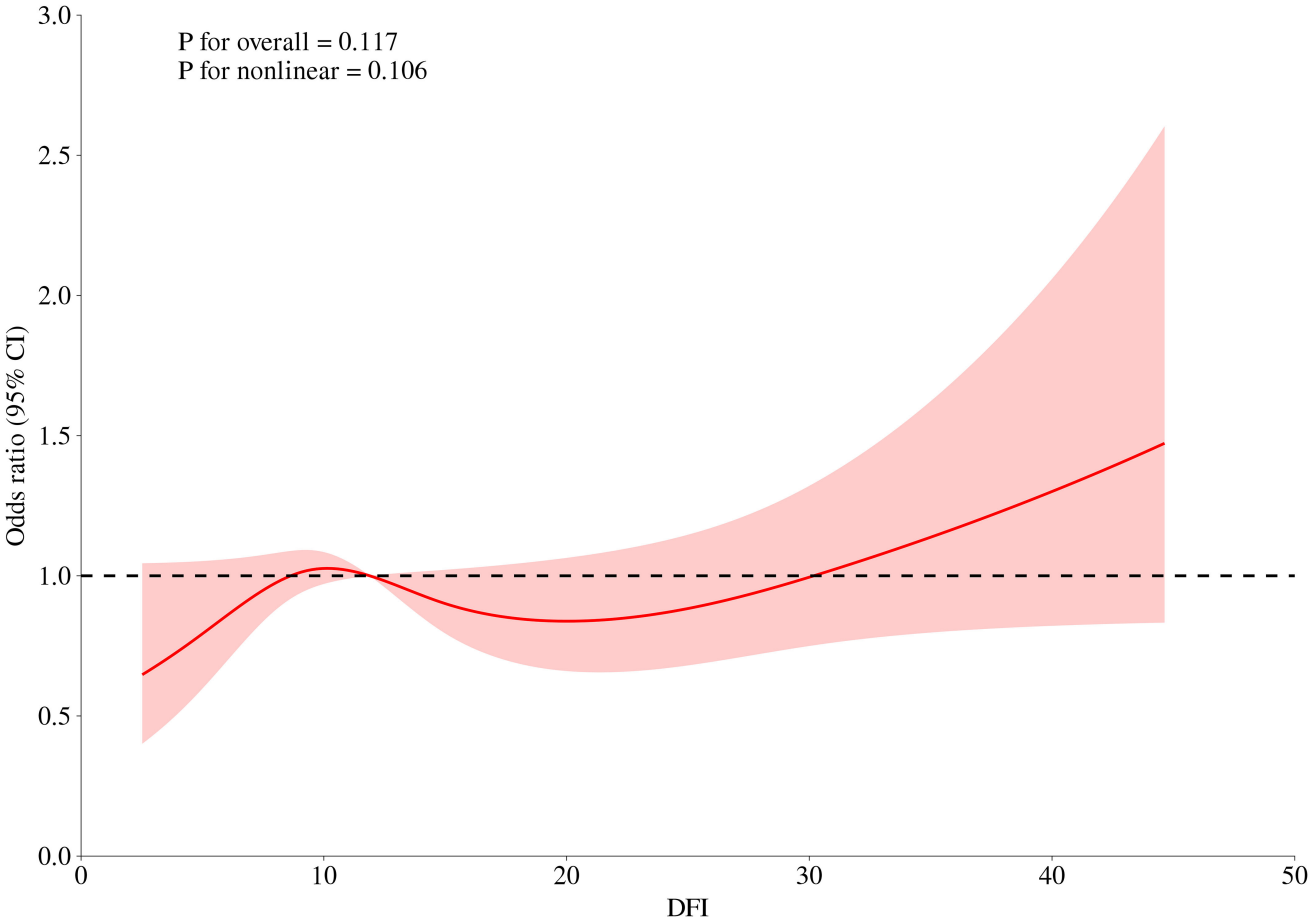
Dose-response curves between DFI and risk of RPL. The DFI was included as a natural cubic spline function, adjusting for age, BMI, education, smoking, drinking, sex frequency, chronic disease, duration of infertility, urogenital infections, varicocele, semen volume, sperm concentration, total motility, progressive motility, TMSC and normal morphology.

The association between abnormal semen parameters and the risk of RPL was further investigated using logistic regression analysis (**Table 4**). No significant associations were observed in either the crude or adjusted models. We further performed multivariate regression models for evaluating the association between sperm DFI and RPL. However, no correlation between DFI and RPL were found (adjusted OR 1.01, 95% CI (1.00-1.03), *P* = 0.09, **Table 5**). RCS was performed to assess the dose-response relationships between RPL and continues DFI (**Figure 1**). The DFI was included as a natural cubic spline function, adjusting for age, BMI, education, smoking, drinking, sex frequency, chronic disease, duration of infertility, urogenital infections, varicocele, semen volume, sperm concentration, total motility, progressive motility, TMSC and normal morphology. No significant non-linear relationships were observed between continuous DFI and the risk of RPL.

**Table 4:** Odds ratios (95% confidence intervals) for abnormal semen parameters in men from couples with RPL.

Semen parameters	Case	
	*OR* (95% CI)	*P*
Crude		
Volume < 1.5 ml	0.72 (0.48-1.08)	0.12
Concentration < 15 million/ml	1.10 (0.72-1.66)	0.67
Progressive motility <32%	0.82 (0.66-1.03)	0.09
Total motility <40%	0.84 (0.68-1.03)	0.10
TMSC < 9 million	0.96 (0.62-1.47)	0.85
Normal morphology < 4%	1.00 (0.67-1.48)	0.99
DFI > 30%	1.24 (0.79-1.92)	0.35
Adjusted		
Volume < 1.5 ml	0.75 (0.49-1.13)	0.17
Concentration < 15 million/ml	1.00 (0.96-1.00)	0.99
Progressive motility <32%	0.80 (0.64-1.01)	0.06
Total motility <40%	0.80 (0.64-1.00)	0.05
TMSC < 9 million	0.89 (0.57-1.38)	0.60
Normal morphology < 4%	0.91 (0.60-1.38)	0.67
DFI > 30%	1.24 (0.78-1.95)	0.36

RPL, recurrent pregnancy loss; TMSC, total motile sperm count; DFI, DNA fragmentation index; OR, odds ratio; CI, confidence interval.

Adjusted model: adjusted for age, BMI, education, smoking, alcohol consumption, chronic diseases, urogenital infections, and varicocele.

**Table 5:** Multivariate regression models for the association between sperm DFI and RPL.

DFI	RPL	
	*OR* (95% CI)	*P*
Crude	1.01(1.00-1.02)	0.23
Adjusted	1.01 (1.00-1.03)	0.09

DFI, DNA fragmentation index; RPL, recurrent pregnancy loss; OR, odds ratio; CI, confidence interval. Adjusted model: adjusted for age, BMI, education, smoking, alcohol consumption, chronic diseases, urogenital infections, varicocele, semen volume, sperm concentration, total motility, progressive motility, TMSC and normal morphology.

## Discussion

Recent studies have suggested a positive correlation between sperm DFI and abnormal pregnancy history. In this study, a total of 1485 men from couples with RPL and those without RPL was recruited, and semen quality, including sperm DFI was evaluated. However, our results indicated that no significant relationship was observed between sperm DFI and the risk of RPL.

Several clinical characteristics, including lifestyle and medical history, have been associated with RPL. A recent study by Esquerre-Lamare et al. recruited 60 participants (33 men from couples with RPL and 27 men from couples with a history of live birth less than the one year) and assessed environmental and family factors as well as semen parameters (21). The results showed that the RPL group were more likely to have a higher male BMI and a family history of infertility. They also found no significant differences in andrological histories and clinical examinations (e.g., cryptorchidism, varicoceles, vas deferens, epididymal position) between cases and controls. However, our study showed that the mean BMI of men did not differ between the study groups, which is consistent with several previously published results (13, 22). In addition, our study found that men from couples with RPL were more likely to have varicocele compared to men from couples without RPL. This finding is consistent with Busnelli et al., who reported a higher prevalence of varicocele in men with RPL compared to the men without RPL(14.9% vs. 8.0%), although this difference did not reach statistical significance (23).

Traditional semen analysis provides a quantitative assessment of sperm concentration, motility and morphology (24). Several studies have shown a trend of low sperm motility and high abnormal sperm morphology in men from couples with RPL (12, 25, 26). However, similar to our findings, many studies that examined the correlation between routine semen parameters and RPL found no significant correlation (27, 28). Since normal sperm genetic material is essential for successful fertilization, as well as embryo and fetal development, sperm DNA integrity is considered a key parameter for evaluating clinical pregnancy outcomes. Several meta-analyses have reported that sperm DFI is associated with RPL (29, 30). However, these meta-analyses may be underpowered due to the limited number of studies and sample sizes included (20). Results from our study are consistent with Zhang et al. that reported no significant difference in DFI between RPL group and control group (27). Additionally, the researchers followed up with the participants for one year and observed a trend toward higher DFI in men from couples with subsequent abnormal pregnancy outcomes compared to those from couples with ongoing pregnancies. This suggests that DFI may be a potential semen parameter for predicting future reproductive outcomes.

A cut-off DFI > 30% was reported as a severe DNA fragmentation in several studies (13, 31, 32). Consistent with other studies, our study also showed a trend of a higher proportion of sperm DFI ≥ 30% in men from the RPL group compared to those from the control group (22, 33). Interestingly, the RCS model indicated that when DFI exceeded 30%, the risk of RPL appeared to increase. Damaged DNA in the sperm may cause embryos to fail in their early stages, contributing to pregnancy loss (34). In addition, sperm DNA contributes to the development of placenta. Fragmented DNA can impair trophoblast development, leading to inadequate placental function, which is a known cause of pregnancy loss (35). Furthermore, DNA fragmentation may disrupt epigenetic marks in sperm, leading to errors in gene expression regulation during embryogenesis. This can result in developmental abnormalities and increase the risk of miscarriage (36). These findings suggest that a threshold DFI, rather than continuous DFI, may be more useful for assessing the correlation between DFI and RPL.

This study has several strengths. First, the relatively large sample size provides substantial statistical power. Additionally, our study included potential confounding factors (e.g. lifestyles and medical history) that have been previously reported to be associated with RPL. However, this study has several limitations. First, the study is limited by its retrospective design and potential selection bias. Second, all participants were recruited from couples undergoing fertility evaluation at a single clinic. Third, although several potential confounders were adjusted for, residual confounding and unknown factors could not be ruled out. It is note that the relationship between sperm DNA fragmentation and recurrent pregnancy loss cannot be fully understood without considering female factors. Maternal age, uterine environment, oocyte quality, and immune response all play significant roles in moderating the impact of DFI on pregnancy outcomes. Forth, the SCSA assay cannot measure DNA strand breaks directly. Finally, different cut-off values for DFI may produce biased results.

In conclusion, our study found no significant relationship between sperm DFI and the risk of RPL. Further prospective studies are needed to investigate the impact of DFI on fertility outcomes in couples experiencing RPL.

## Data Availability

The raw data supporting the conclusions of this article will be made available by the authors, without undue reservation.
